# YAP/TAZ‐TEAD signalling axis: A new therapeutic target in malignant pleural mesothelioma

**DOI:** 10.1111/jcmm.18330

**Published:** 2024-04-12

**Authors:** Kostas A. Papavassiliou, Amalia A. Sofianidi, Athanasios G. Papavassiliou

**Affiliations:** ^1^ First University Department of Respiratory Medicine, ‘Sotiria’ Hospital, Medical School National and Kapodistrian University of Athens Athens Greece; ^2^ Department of Biological Chemistry, Medical School National and Kapodistrian University of Athens Athens Greece

**Keywords:** Hippo signalling, malignant pleural mesothelioma, TEAD, TEAD inhibitor, YAP/TAZ

## Abstract

The Hippo signalling pathway, a highly conserved signalling cassette, regulates organ size by controlling cell growth, apoptosis and stem cell self‐renewal. The tumourigenic potential of this pathway is largely attributed to the activity of YAP/TAZ, which activate the TEAD1‐4 transcription factors, leading to the expression of genes involved in cell proliferation and suppression of cell death. Aberrant regulation of the YAP/TAZ‐TEAD signalling axis is commonly observed in malignant pleural mesothelioma (MPM), an insidious neoplasm of the pleural tissue that lines the chest cavity and covers the lungs with poor prognosis. Given the limited effectiveness of current treatments, targeting the YAP/TAZ‐TEAD signalling cascade has emerged as a promising therapeutic strategy in MPM. Several inhibitors of the YAP/TAZ‐TEAD signalling axis are presently undergoing clinical development, with the goal of advancing them to clinical use in the near future.

## 1 YAP/TAZ BIOLOGY: STRUCTURE/FUNCTION RELATIONSHIPS

The yes‐associated protein (YAP) and the transcriptional coactivator with PDZ‐binding motif (TAZ) are transcriptional cofactors, integral components of the Hippo signalling pathway. In humans, the *YAP1* gene is located on chromosome 11q22 and encodes the 65‐kDa YAP protein. YAP comprises several functional domains, including a transcriptional enhanced associate domain (TEAD)‐binding (TB) region, two WW domains, an SH3‐binding motif, a coiled‐coil domain, a transcription activation domain, an N‐terminal proline‐rich domain and a C‐terminal PDZ‐binding motif. There are eight different isoforms of the *YAP* gene, which are classified into YAP1 and YAP2 subgroups and differ in the WW domain. The *TAZ* gene is located on chromosome 3q23‐q24 and encodes the 43‐kDa TAZ protein, which is structurally similar to YAP but has only one WW domain and lacks the SH3‐binding motif and the proline‐rich domain.^1^, ^2^

YAP and TAZ are transcriptional coactivators whose localization alternates between the cytoplasm and the nucleus. Their WW domains interact with PPXY motifs of various transcription factors, and their TB region serves as an interaction site with members of the TEAD family of transcription factors (TEAD1‐4), triggering the expression of genes associated with malignant transformation in certain cancer types. YAP and TAZ activity is also regulated by members of the 14‐3‐3 family of phosphoprotein‐binding proteins.^3^ They share various transcription factors, but they also maintain their own target transcription factors, such as ErbB4 and p73 for YAP and peroxisome proliferator‐activated receptor gamma (PPARγ), paired box gene 3 (Pax3), T‐box 5 (TBX5) and thyroid transcription factor‐1 (TTF‐1) for TAZ.^1^

## 2 YAP/TAZ–TEAD: SIGNALLING MECHANISM

**FIGURE 1:**
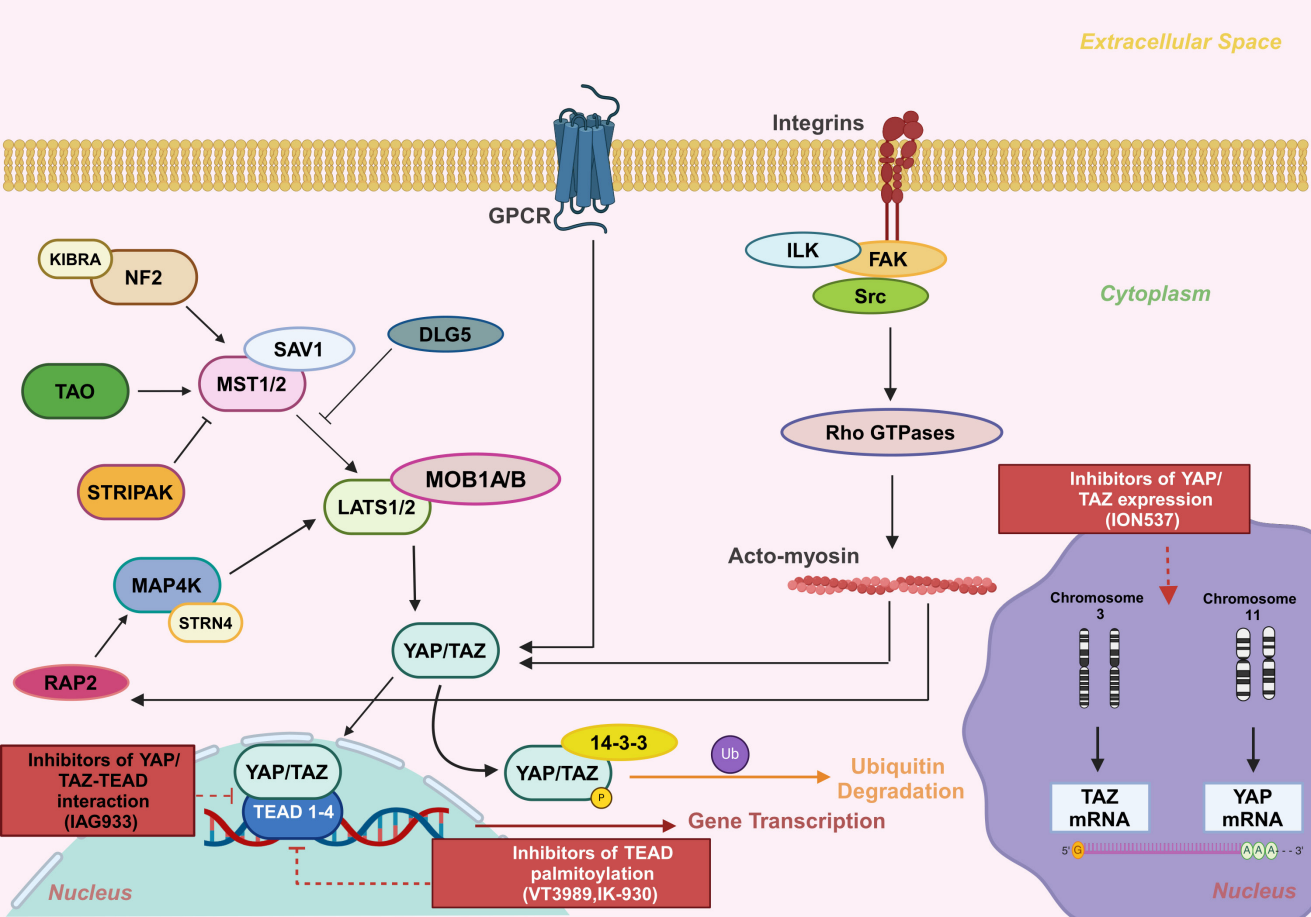
Overview of the YAP/TAZ‐TEAD signalling axis. The core component of the Hippo pathway consists of the kinases MST1/2 and LATS1/2, which phosphorylate the YAP/TAZ complex. Once phosphorylated, YAP/TAZ interact with the 14‐3‐3 protein and undergo ubiquitin‐mediated proteasomal degradation. Unphosphorylated YAP/TAZ translocate to the nucleus, bind to TEAD1‐4 transcription factors and regulate the expression of target genes responsible for cell proliferation and inhibition of cell death. Several factors influence the Hippo pathway: (i) KIBRA‐NF2 and TAO activate MST1/2, while DLG5 and STRIPAK inhibit MST1/2. At low intracellular tension, RAP2 potentiates MAP4K, which, in turn, activates LATS1/2. (ii) GPCRs either activate or deactivate the YAP/TAZ complex, depending on the type of the GPCR. (iii) mechanotransduction also regulates YAP/TAZ activity. Integrins are connected to the ILK/FAK/Src pathway and potentiate YAP/TAZ through Rho GTPases and acto‐myosin. Ιnhibitors of the YAP/TAZ‐TEAD signalling cascade which are currently in phase I clinical trials are depicted in rectangulars. DLG5, discs large homologue 5; FAK, focal adhesion kinase; GPCR, G protein‐coupled receptor; ILK, integrin‐linked kinase; KIBRA, kidney and brain protein; LATS1/2, large tumour suppressor kinase 1/2; MAP4K, mitogen‐activated protein kinase kinase kinase kinase; MOB1A/B, MOB kinase activator 1A/1B; MST1/2, STE20‐like protein kinase 1/2; NF2, neurofibromatosis type 2; RAP2, RAS‐related GTPase; STRIPAK, striatin‐interacting phosphatase and kinase complex; TAO, thousand‐and‐one amino acid kinases; TAZ, transcriptional coactivator with PDZ‐binding motif; TEAD, transcriptional enhanced associate domain; YAP, yes‐associated protein. This figure was created based on the tools provided by Biorender.com (https://biorender.com/).

The Hippo signalling pathway is an evolutionarily conserved pathway that controls tissue growth and homeostasis by regulating cell proliferation, apoptosis and stem cell self‐renewal.^4^ It comprises three main units: (i) upstream regulatory elements (neurofibromatosis type 2 (NF2)), (ii) a central regulatory serine–threonine kinase unit (STE20‐like protein kinase 1/2 (MST1/2) and large tumour suppressor 1/2 (LATS1/2)) and (iii) a downstream transcriptional effector unit (YAP/TAZ)^1^ (Figure 1). MST1/2 and its binding partner Salvador (SAV1) potentiate LATS1/2 along with its adaptor partner MOB kinase activator 1A/1B (MOB1A/B).^5^, ^6^ The activated LATS1/2‐MOB1A/B complex phosphorylates YAP and TAZ to initiate the Hippo cascade.^5^ Moreover, the junction‐associated angiomotin (AMOT) scaffold family of proteins binds to LATS1 and SAV1‐MST1, thereby connecting YAP to LATS1.^7^ Once phosphorylated, YAP/TAZ interact with the 14‐3‐3 protein, leading to the retention of YAP/TAZ in the cytoplasm and eventually ubiquitin‐mediated proteasomal degradation.^1^ When the Hippo pathway is deactivated, unphosphorylated YAP/TAZ translocate to the nucleus. There, they bind to TEAD1‐4 transcription factors (Figure 1), modulating the expression of target genes responsible for cell proliferation and inhibition of cell death. These mechanisms support the tumourigenic potential of YAP/TAZ.^8^

Multiple upstream regulators of the Hippo cascade have been described. Among these is the tumour suppressor protein NF2 (also known as Merlin), which forms a complex with kidney and brain protein (KIBRA; also known as WWC1). Together, they activate MST1/2 or recruit LATS1/2 for activation by MST1/2, ultimately phosphorylating the YAP/TAZ complex.^9^ The striatin‐interacting phosphatase and kinase (STRIPAK) complex dephosphorylates MST1/2, leading to YAP/TAZ activation.^10^ Thousand‐and‐one amino acid kinases (TAO) activate MST1/2 through direct phosphorylation, thus inhibiting the transcriptional function of YAP/TAZ^11^ (Figure 1). The mitogen‐activated kinase kinase kinase kinase (MAP4K) family of kinases contributes significantly to the activation of the Hippo cascade, by directly phosphorylating and activating LATS1/2 (Figure 1). Additionally, a STRIPAK complex component, STRN4, regulates YAP through MAP4Ks in a similar way as STRIPAK complex interacts with MST1/2^12^ (Figure 1). Other upstream regulators of the Hippo cascade are the G protein‐coupled receptors (GPCRs) (Figure 1). GPCRs linked with G12/13, Gq/11 or Gi/o proteins, such as lysophosphatidic acid (LPA) and thrombin receptors, trigger the activation of YAP/TAZ. Conversely, GPCRs associated with Gs proteins, such as epinephrine and glucagon receptors, impede the activity of YAP/TAZ.^13^

Mechanotransduction, the exchange of physical forces between cells and their extracellular matrix (ECM), is a critical factor regulating YAP/TAZ activity. This exchange is primarily mediated by integrins, which are connected on their cytoplasmic side with the F‐actin cytoskeleton through focal adhesions, in a manner involving the integrin‐linked kinase (ILK), focal adhesion kinase (FAK) and Src proteins. Extracellular mechanical cues force the cell to increase its contractility through downstream effectors of the ILK/FAK/Src pathway, namely Rho GTPases (such as Rho or Rac1), Rho‐associated protein kinase (ROCK) and acto‐myosin (Figure 1). YAP/TAZ nuclear localization and activation eventually occurs.^14^, ^15^ In turn, acto‐myosin contractility can affect LATS1/2 activity through the GTPase RAP2^16^ (Figure 1). Regarding cell–cell adhesion molecules, α‐catenin is attached to the cytoplasmic domain of E‐cadherin. In the presence of 14‐3‐3, α‐catenin binds to phosphorylated YAP, preventing YAP activation and nuclear translocation.^17^ Cell–cell adhesion molecules play a role in establishing cell polarity and forming adherens junctions. Adherens junctions, in turn, modulate the Hippo pathway by impacting the localization and activity of components such as NF2.^18^ Discs large homologue 5 (DLG5) is a protein member of the membrane‐associated guanylate kinase (MAGUK) complex, which consists of scaffold molecules for protein complexes containing various receptors and signalling elements on the cell membrane. DLG5 directly interacts with MST1/2 to negatively regulate the activity of the Hippo pathway^19^ (Figure 1). Epithelial–mesenchymal transition (EMT) is characterized by loss of cell polarity, disruption of cell–cell junctions and cytoskeletal remodelling. EMT is implicated in carcinogenesis by activating YAP and TAZ and promoting tumour survival and progression.^20^

Nuclear YAP/TAZ accumulation above a critical threshold is linked to various cancer hallmarks of different cancer types, including malignant pleural mesothelioma (MPM). These encompass traits such as cell proliferation, phenotypic plasticity, resistance to drugs, acquisition of metastatic potential and modulation of the tumour microenvironment through the control of stromal cells, inflammation, senescence, immunity and angiogenesis.^21^

## 3 TARGETING THE YAP/TAZ‐TEAD SIGNALLING AXIS IN MPM

Malignant pleural mesothelioma is an aggressive asbestos‐associated thoracic tumour originating from pleural mesothelial cells with a poor prognosis (median survival of MPM patients is a mere 8–14 months), due to late‐stage diagnosis and a highly infiltrative phenotype. NF2, a major upstream activator of the Hippo pathway, is commonly mutated in MPM, with 30%–40% of cases exhibiting NF2 inactivation.^22^ Notwithstanding the progress in diagnostic tools and biomolecular research, therapeutic options for the management of MPM are still limited; therefore, novel therapeutic targets are currently being explored. The overexpression of YAP/TAZ is associated with oncotherapy resistance, leading to the ineffectiveness of the available treatment approaches.^23^ On this basis, the YAP/TAZ‐TEAD signalling axis was recently introduced as a novel therapeutic target in MPM.

YAP/TAZ have been characterized as challenging targets for drug development. Nevertheless, there have been efforts to experimentally decrease their expression using RNA‐interference (RNAi) methods. ION537, an antisense oligonucleotide (ASO) targeting YAP1 mRNA effectively hindered YAP expression in tumour xenografts^24^ and is currently being evaluated in a phase I clinical trial (NCT04659096), enrolling patients with solid malignancies.

TEAD activity depends on palmitoylation, a highly conserved process that involves the attachment of palmitate onto cysteine residues via a thioester linkage.^25^ Using a mesothelioma xenograft model, Tang et al. targeted palmitoylation with small‐molecule compounds and demonstrated that *NF2*‐deficient cancer cells exhibit sensitivity to inhibition of TEAD palmitoylation.^26^ VT3989 is an orally administered, highly potent and selective inhibitor of TEAD palmitoylation, which blocks YAP function and has shown promising preclinical activity. The first‐in‐human phase I trial (NCT04665206) of VT3989 enrolled 67 patients who had progressed on prior chemotherapy regimens, 29 of whom had MPM. VT3989 was found to be safe and well tolerated without dose‐limiting toxicities. The most common adverse events were proteinuria, albuminuria and peripheral edema, primarily when the drug was administered in a continuous schedule. Seven patients (six with refractory malignant mesothelioma pleural or non‐pleural) achieved RECIST v1.1 partial responses.^27^ Another oral small‐molecule inhibitor of TEAD palmitoylation, which is presently in phase I clinical trial evaluation (NCT05228015), is IK‐930. In preclinical studies, IK‐930 exhibited antitumour effects in mouse xenograft models featuring genetic alterations in the Hippo pathway. Currently, it is being assessed as an oral treatment option for patients with advanced solid tumours.^28^

There have also been efforts to target YAP/TAZ interaction with TEAD, with the pioneer drug being verteporfin, which however displayed YAP‐independent cytotoxic effects.^29^ IAG933, a potent and direct small‐molecule inhibitor that disrupts the interaction between YAP and TEAD proteins, is currently in a phase I clinical trial (NCT04857372) in patients with mesothelioma, *NF2*/*LATS1*/*2*‐mutated tumours and tumours harbouring functional YAP/TAZ fusions (i.e., YAP/TAZ hybrids that hyperactivate a TEAD‐based transcriptome). In preclinical xenograft and primary‐tumour derived MPM models, IAG933 demonstrated robust antitumour efficacy.^30^ Table 1 and Figure 1 present the inhibitors of the YAP/TAZ‐TEAD axis that are currently being evaluated in clinical trials.

**TABLE 1:** Inhibitors of the YAP/TAZ‐TEAD axis currently in clinical trials.

Target	Inhibitor name	Efficacy
YAP/TAZ expression	ION537	Ongoing phase I clinical trial (NCT04659096) in pts with advanced solid tumors^24^
TEAD palmitoylation	VT3989	Phase I clinical trial (NCT04665206): Enrolled 67 pts (29 with MPM). Median prior therapies 3 (range 0–8) Most common TRAEs: proteinuria, albuminuria and peripheral edema. 7 G3 TRAEs (fatigue, ↑ALT & AST, dehydration, dyspnea, hypotension, peripheral edema) and 1 G4 TRAE (cardiomyopathy) Results: 6 refractory MM achieved RECIST v1.1 PRs. 3 PRs in MM pts are ongoing up to 18+ months. Clinical benefit response rate (PR + SD >8 weeks, per protocol) in MM pts is 57%^27^
	IK‐930	Ongoing phase I clinical trial (NCT05228015) in pts with advanced solid tumors^28^
YAP/TAZ interaction with TEAD	IAG933	Ongoing phase I clinical trial (NCT04857372) in pts with MM, *NF2*/*LATS1*/*2*‐mutated tumours and tumours with functional YAP/TAZ fusions^30^

Abbreviations: ALT, alanine aminotransferase; AST, aspartate aminotransferase; G, grade; MM, malignant mesothelioma; MPM, malignant pleural mesothelioma; PR, partial response; pts, patients; RECIST, response evaluation criteria in solid tumours; SD, stable disease; TRAEs, treatment‐related adverse events.

In the preclinical setting, SWTX‐143 is a YAP/TAZ‐TEAD axis inhibitor which binds to the palmitoylation pocket of all four TEAD isoforms and causes irreversible TEAD inhibition. In subcutaneous xenograft models with human cells and in an orthotopic mesothelioma mouse model, it was demonstrated that SWTX‐143 causes strong mesothelioma regression.^31^ K‐975 is another potent, selective and orally active TEAD inhibitor, with a strong inhibitory effect against protein–protein interactions between YAP1/TAZ and TEAD. It exhibited strong antitumour activity in preclinical mesothelioma models, but it was associated with renal toxicity which might present challenges in clinical application.^32^ Lastly, JM7 was found to preclinically inhibit YAP transcriptional reporter activity in *NF2*‐mutant mesothelioma cells. JM7 is novel small‐molecule inhibitor of YAP activity, which hampers TEAD palmitoylation alongside YAP target‐gene expression, without affecting YAP/TEAD localization. Since YAP activity in cancer cells and immune cells interferes with immunotherapy,^33^ JM7 could be combined with immunotherapeutic agents in clinical models in the future.^34^

## 4 PERSPECTIVES – OUTLOOK

Future steps in the field could be oriented towards targeting other molecules of the Hippo cascade. Potential therapeutic agents targeting YAP/TAZ include bromodomain and extra‐terminal motif (BET) inhibitors, which focus on bromodomain‐containing protein 4 (BRD4) and its related proteins, as YAP/TAZ are known to engage BRD4 on chromatin. Statins impede YAP nuclear translocation and augment sensitivity to specific cancer medications. Molecules interfering with the activity of Src and its family members (e.g., dasatinib) have also exhibited YAP/TAZ inhibition in vitro and in vivo. Rho GTPases and ROCK are also appealing targets.^15^ Nonetheless, accumulating evidence suggests that YAP/TAZ may also have a tumour‐suppressive role depending on the context. Thus, careful consideration is warranted when exploring Hippo signalling as a target in future clinical trials.^35^

Interestingly, it has been shown that sirtuin 1 (SIRT1), a NAD^+^‐dependent protein deacetylase, deacetylates YAP2 in hepatocellular carcinoma (HCC) cells and SIRT1‐mediated YAP deacetylation increases the YAP2‐TEAD4 association, leading to YAP2‐TEAD4 transcriptional activation and upregulated cell growth in HCC cells.^36^ In this vein, an increasing amount of preclinical data highlights the effectiveness of histone deacetylase inhibition in MPM cell lines and mouse xenograft models,^37^ suggesting that the combinatorial use of histone deacetylase inhibitors (HDCi), for example, depsipeptide, a HDACi exhibiting antitumour effects against several types of solid tumours,^38^ with immunotherapy may provide an additional strategy towards formulating more effective therapeutic regimens in patients with MPM. However, as emphasized for other solid tumour types, such as breast carcinomas with malignant‐appearing microcalcifications on mammography,^39^, ^40^ stage‐specific biomarker ‘signatures’/gene expression profiling and immune system status should also be taken into consideration in designing tailored combinatorial therapies for MPM patients.

## 5 CONCLUSION

The YAP/TAZ‐TEAD signalling axis is often dysregulated in multiple tumour subtypes, including MPM. Allosteric inhibitors that disrupt the YAP–TEAD interaction have shown promising antitumour efficacy mainly in the preclinical setting. Various broadly acting TEAD inhibitors are presently being developed aiming at advancing them to clinical use as monotherapy or in combination with the existing treatment options in the years to come. The outcomes of the ongoing clinical trials will provide insights into the effectiveness of these inhibitors in managing MPM, potentially opening new avenues for improved overall survival of cancer patients.

## AUTHOR CONTRIBUTIONS

**Kostas A. Papavassiliou:** Conceptualization (lead); data curation (equal); writing – original draft (lead). **Amalia A. Sofianidi:** Data curation (lead); writing – original draft (lead). **Athanasios G. Papavassiliou:** Conceptualization (lead); supervision (lead); writing – review and editing (lead).

## CONFLICT OF INTEREST STATEMENT

The authors declare no competing financial or non‐financial interests.

## Data Availability

Data sharing not applicable – no new data generated.

## References

[1] Liu H , Du S , Lei T , et al. Multifaceted regulation and functions of YAP/TAZ in tumors (review). Oncol Rep. 2018;40(1):16‐28. doi:10.3892/or.2018.6423 29749524 PMC6059739

[2] Ortega Á , Vera I , Diaz M , et al. The YAP/TAZ signaling pathway in the tumor microenvironment and carcinogenesis: current knowledge and therapeutic promises. Int J Mol Sci. 2021;23(1):430. doi:10.3390/ijms23010430 35008857 PMC8745604

[3] Pocaterra A , Romani P , Dupont S . YAP/TAZ functions and their regulation at a glance. J Cell Sci. 2020;133(2):jcs230425. doi:10.1242/jcs.230425 31996398

[4] Fu M , Hu Y , Lan T , Guan KL , Luo T , Luo M . The hippo signalling pathway and its implications in human health and diseases. Signal Transduct Target Ther. 2022;7(1):376. doi:10.1038/s41392-022-01191-9 36347846 PMC9643504

[5] Boopathy GTK , Hong W . Role of hippo pathway‐YAP/TAZ signaling in angiogenesis. Front Cell Dev Biol. 2019;7:49. doi:10.3389/fcell.2019.00049 31024911 PMC6468149

[6] Cunningham R , Hansen CG . The hippo pathway in cancer: YAP/TAZ and TEAD as therapeutic targets in cancer. Clin Sci (Lond). 2022;136(3):197‐222. doi:10.1042/CS20201474 35119068 PMC8819670

[7] Mana‐Capelli S , McCollum D . Angiomotins stimulate LATS kinase autophosphorylation and act as scaffolds that promote hippo signaling. J Biol Chem. 2018;293(47):18230‐18241. doi:10.1074/jbc.RA118.004187 30266805 PMC6254346

[8] Totaro A , Panciera T , Piccolo S . YAP/TAZ upstream signals and downstream responses. Nat Cell Biol. 2018;20(8):888‐899. doi:10.1038/s41556-018-0142-z 30050119 PMC6186418

[9] Sabra H , Brunner M , Mandati V , et al. β1 integrin–dependent Rac/group I PAK signaling mediates YAP activation of yes‐associated protein 1 (YAP1) via NF2/merlin. J Biol Chem. 2017;292(47):19179‐19197. doi:10.1074/jbc.M117.808063 28972170 PMC5702661

[10] Zheng Y , Liu B , Wang L , Lei H , Pulgar Prieto KD , Pan D . Homeostatic control of Hpo/MST kinase activity through autophosphorylation‐dependent recruitment of the STRIPAK PP2A phosphatase complex. Cell Rep. 2017;21(12):3612‐3623. doi:10.1016/j.celrep.2017.11.076 29262338 PMC5741103

[11] Yang Y , Zhou H , Huang X , et al. Innate immune and proinflammatory signals activate the hippo pathway via a Tak1‐STRIPAK‐Tao axis. Nat Commun. 2024;15(1):145. doi:10.1038/s41467-023-44542-y 38168080 PMC10761881

[12] Seo G , Han H , Vargas RE , Yang B , Li X , Wang W . MAP4K interactome reveals STRN4 as a key STRIPAK complex component in hippo pathway regulation. Cell Rep. 2020;32(1):107860. doi:10.1016/j.celrep.2020.107860 32640226 PMC7382313

[13] Luo J , Yu FX . GPCR‐hippo signaling in cancer. Cancer Cells. 2019;8(5):426. doi:10.3390/cells8050426 PMC6563442 31072060

[14] Wolfenson H , Yang B , Sheetz MP . Steps in Mechanotransduction pathways that control cell morphology. Annu Rev Physiol. 2019;81:585‐605. doi:10.1146/annurev-physiol-021317-121245 30403543 PMC7476682

[15] Piccolo S , Panciera T , Contessotto P , Cordenonsi M . YAP/TAZ as master regulators in cancer: modulation, function and therapeutic approaches. Nat Cancer. 2023;4(1):9‐26. doi:10.1038/s43018-022-00473-z 36564601 PMC7614914

[16] Meng Z , Qiu Y , Lin KC , et al. RAP2 mediates mechanoresponses of the hippo pathway. Nature. 2018;560(7720):655‐660. doi:10.1038/s41586-018-0444-0 30135582 PMC6128698

[17] Chang YC , Wu JW , Wang CW , Jang ACC . Hippo signaling‐mediated Mechanotransduction in cell movement and cancer metastasis. Front Mol Biosci. 2020;6:157. doi:10.3389/fmolb.2019.00157 32118029 PMC7025494

[18] Ahmad US , Uttagomol J , Wan H . The regulation of the hippo pathway by intercellular junction proteins. Life (Basel). 2022;12(11):1792. doi:10.3390/life12111792 36362947 PMC9696951

[19] Song X‐Q , Li Q , Zhang J . A double‐edged sword: DLG5 in diseases. Biomed Pharmacother. 2023;162:114611. doi:10.1016/j.biopha.2023.114611 37001186

[20] Akrida I , Bravou V , Papadaki H . The deadly cross‐talk between hippo pathway and epithelial–mesenchymal transition (EMT) in cancer. Mol Biol Rep. 2022;49(10):10065‐10076. doi:10.1007/s11033-022-07590-z 35604626

[21] Hanahan D . Hallmarks of cancer: new dimensions. Cancer Discov. 2022;12(1):31‐46. doi:10.1158/2159-8290.CD-21-1059 35022204

[22] Hiltbrunner S , Fleischmann Z , Sokol ES , Zoche M , Felley‐Bosco E , Curioni‐Fontecedro A . Genomic landscape of pleural and peritoneal mesothelioma tumours. Br J Cancer. 2022;127(11):1997‐2005. doi:10.1038/s41416-022-01979-0 36138075 PMC9681755

[23] Nguyen CDK , Yi C . YAP/TAZ signaling and resistance to cancer therapy. Trends Cancer. 2019;5(5):283‐296. doi:10.1016/j.trecan.2019.02.010 31174841 PMC6557283

[24] Macleod AR . The discovery and characterization of ION‐537: a next generation antisense oligonucleotide inhibitor of YAP1 in preclinical cancer models. Cancer Res. 2021;81(13_Supplement):ND11. doi:10.1158/1538-7445.AM2021-ND11

[25] Kim NG , Gumbiner BM . Cell contact and Nf2/Merlin‐dependent regulation of TEAD palmitoylation and activity. Proc Natl Acad Sci USA. 2019;116(20):9877‐9882. doi:10.1073/pnas.1819400116 31043565 PMC6525549

[26] Tang TT , Konradi AW , Feng Y , et al. Small molecule inhibitors of TEAD auto‐palmitoylation selectively inhibit proliferation and tumor growth of *NF2*‐deficient mesothelioma. Mol Cancer Ther. 2021;20(6):986‐998. doi:10.1158/1535-7163.MCT-20-0717 33850002

[27] Yap TA , Kwiatkowski DJ , Desai J , et al. First‐in‐class, first‐in‐human phase 1 trial of VT3989, an inhibitor of yes‐associated protein (YAP)/transcriptional enhancer activator domain (TEAD), in patients (pts) with advanced solid tumors enriched for malignant mesothelioma and other tumors with neurofibromatosis 2 (NF2) mutations. Cancer Res. 2023;83(8_Supplement):CT006. doi:10.1158/1538-7445.AM2023-CT006

[28] Tolcher AW , Lakhani NJ , McKean M , et al. A phase 1, first‐in‐human study of IK‐930, an oral TEAD inhibitor targeting the hippo pathway in subjects with advanced solid tumors. J Clin Oncol. 2022;40(16_suppl):TPS3168. doi:10.1200/JCO.2022.40.16_suppl.TPS3168

[29] Liu‐Chittenden Y , Huang B , Shim JS , et al. Genetic and pharmacological disruption of the TEAD‐YAP complex suppresses the oncogenic activity of YAP. Genes Dev. 2012;26(12):1300‐1305. doi:10.1101/gad.192856.112 22677547 PMC3387657

[30] Schmelzle T , Chapeau E , Bauer D , et al. IAG933, a selective and orally efficacious YAP1/WWTR1(TAZ)‐panTEAD protein‐protein interaction inhibitor with pre‐clinical activity in monotherapy and combinations. Cancer Res. 2023;83(8_Supplement):LB319. doi:10.1158/1538-7445.AM2023-LB319

[31] Hillen H , Candi A , Vanderhoydonck B , et al. A novel irreversible TEAD inhibitor, SWTX‐143, blocks hippo pathway transcriptional output and causes tumor regression in preclinical mesothelioma models. Mol Cancer Ther. 2024;23(1):3‐13. doi:10.1158/1535-7163.MCT-22-0681 37748190

[32] Kaneda A , Seike T , Uemori T , et al. Discovery of a *first‐in‐class* TEAD inhibitor which directly inhibits YAP/TAZ‐TEAD protein‐protein interaction and shows a potent anti‐tumor effect in malignant pleural mesothelioma. Cancer Res. 2019;79(13_Supplement):3086. doi:10.1158/1538-7445.AM2019-3086

[33] Yu M , Peng Z , Qin M , et al. Interferon‐γ induces tumor resistance to anti‐PD‐1 immunotherapy by promoting YAP phase separation. Mol Cell. 2021;81(6):1216‐1230.e9. doi:10.1016/j.molcel.2021.01.010 33606996

[34] Gridnev A , Maity S , Misra JR . Structure‐based discovery of a novel small‐molecule inhibitor of TEAD palmitoylation with anticancer activity. Front Oncol. 2022;12:1021823. doi:10.3389/fonc.2022.1021823 36523977 PMC9745137

[35] Luo J , Deng L , Zou H , et al. New insights into the ambivalent role of YAP/TAZ in human cancers. J Exp Clin Cancer Res. 2023;42(1):130. doi:10.1186/s13046-023-02704-2 37211598 PMC10201886

[36] Mao B , Hu F , Cheng J , et al. SIRT1 regulates YAP2‐mediated cell proliferation and chemoresistance in hepatocellular carcinoma. Oncogene. 2014;33(11):1468‐1474. doi:10.1038/onc.2013.88 23542177

[37] Paik PK , Krug LM . Histone deacetylase inhibitors in malignant pleural mesothelioma: preclinical rationale and clinical trials. J Thorac Oncol. 2010;5(2):275‐279. doi:10.1097/JTO.0b013e3181c5e366 20035240 PMC4052955

[38] Konstantinopoulos PA , Vandoros GP , Papavassiliou AG . FK228 (depsipeptide): a HDAC inhibitor with pleiotropic antitumor activities. Cancer Chemother Pharmacol. 2006;58(5):711‐715. doi:10.1007/s00280-005-0182-5 16435156

[39] Karamouzis MV , Likaki‐Karatza E , Ravazoula P , et al. Non‐palpable breast carcinomas: correlation of mammographically detected malignant‐appearing microcalcifications and molecular prognostic factors. Int J Cancer. 2002;102(1):86‐90. doi:10.1002/ijc.10654 12353238

[40] Shin SU , Lee J , Kim JH , et al. Gene expression profiling of calcifications in breast cancer. Sci Rep. 2017;7:11427. doi:10.1038/s41598-017-11331-9 28900139 PMC5595962

